# Outer membrane vesicles secreted by *Bacteroides fragilis* inhibit CFTR chloride secretion by human colon organoids

**DOI:** 10.1128/iai.00129-26

**Published:** 2026-05-29

**Authors:** Roxanna Barnaby, Amanda Nymon, Paige Salerno, Carolyn T. Roche, Young Ah Goo, Byoung-Kyu Cho, Timothy B. Gardner, Zdenek Svindrych, Douglas J. Taatjes, Thomas H. Hampton, Benjamin Ross, Bruce A. Stanton

**Affiliations:** 1 Department of Microbiology and Immunology, Geisel School of Medicine at Dartmouth, Hanover, New Hampshire, USA; 2 Mass Spectrometry Technology Access Center, McDonnell Genome Institute, Washington University School of Medicine 12275, St. Louis, Missouri, USA; 3 Center for Digestive Health, Dartmouth Hitchcock Medical Center 22916, Lebanon, New Hampshire, USA; 4 Department of Biochemistry and Cell Biology, Geisel School of Medicine at Dartmouth, Hanover, USA; 5 Department of Pathology and Laboratory Medicine, University of Vermont 2092, Burlington, Vermont, USA; Helmholtz-Zentrum fur Infektionsforschung GmbH, Braunschweig, Germany

**Keywords:** *Bacteroides fragilis*, outer membrane vesicles, CFTR, transepithelial resistance, colon organoids

## Abstract

The goals of this study were to develop a model to study host-pathogen interactions in primary human colon organoids and to test the hypothesis that *Bacteroides fragilis* toxin (BFT-2) secreted in outer membrane vesicles (OMVs) modulates mucosal immunity and CFTR Cl⁻ secretion. Since *Bacteroides* species reside in mucus, OMVs are likely to represent a mechanism of communication between *Bacteroides* and the host. Two strains of *Bacteroides* were studied: enterotoxigenic *Bacteroides fragilis* (ETBF), which produces BFT-2, and the non-toxigenic *Bacteroides fragilis* strain NCTC 9343 (NTBF), which does not produce BFT-2. We also utilized two additional strains of *Bacteroides fragilis*: one in which *bft*-2 was knocked out (ETBF Δ*bft*) and one that was engineered to contain *bft-2* (NTBF+*bft*). We report that *Bacteroides fragilis* OMVs reduced CFTR Cl⁻ secretion, but had no effect on tight junction or cell adhesion proteins, transepithelial resistance (TER), or cytokine secretion by primary human colon organoids. NTBF OMVs containing BFT-2 were more effective in reducing CFTR Cl*^-^* secretion than NTBF lacking BFT-2. By contrast, deletion of *bft* in ETBF did not have a significant effect on CFTR Cl⁻ secretion compared to ETBF. We conclude that OMVs secreted by *Bacteroides* can be an important mechanism of host-pathogen interactions in the colon by reducing CFTR Cl⁻ secretion, and that the effect of BFT-2 on CFTR Cl⁻ secretion is dependent on the strain of *Bacteroides fragilis*.

## INTRODUCTION

The human gastrointestinal tract is colonized by ~100 trillion bacteria, including several members of the genus *Bacteroides*, which comprise approximately 30% of the microbiota (1–5). Although *Bacteroides fragilis* (*B. fragilis*) is one of the least abundant species, enterotoxigenic *B. fragilis* (ETBF) is a pathogenic bacterium that induces diarrhea, colitis, and tumor formation through the secretion of *Bacteroides* fragilis toxin (BFT-2), a metalloprotease (3, 6–8). Many, but not all, studies on colon cell lines have shown that recombinant BFT-2 rapidly (~1–3 h) causes cell damage and disrupts cell adhesion by cleaving E-cadherin, a cell adhesion protein, leading to an increase in epithelial paracellular permeability, IL-8 secretion (3, 9–12), fluid secretion driven by CFTR Cl⁻ channels (13, 14), and diarrhea (15–17). However, in some studies on colon cell lines, E-cadherin remained unchanged after exposure to ETBF supernatant (9). The effect of non-toxigenic *Bacteroides fragilis* (NTBF) and ETBF OMVs on IL-8 secretion and E-cadherin in colon cell lines is dose-, strain-, and time-dependent (9, 12, 18). It has been suggested that the BFT-2-induced increase in permeability leads to paracellular movement of bacteria and bacterial products from the lumen of the colon to the submucosal layer and blood, an effect that can train the immune system (2). Recombinant BFT-2 applied to the apical side of HT 29/C1, HT-29, T84, CMT93, or MDCK cell lines decreases transepithelial resistance (TER) and cleaves E-cadherin (9, 11, 15, 17, 19, 20). One study on the T84 colon cell line reported no effect of BFT-2 on short-circuit current but did report that BFT-2 reduced TER (20).

Many Gram-negative bacteria that reside in the mucus lining of the colon secrete outer membrane vesicles (OMVs) (12, 21–26) that stimulate inflammation and decrease ion transport, including CFTR Cl⁻ secretion by epithelial cell lines (21–23, 27). Therefore, it is likely that communication between *B. fragilis* and human colon cells is mediated by the secretion of OMVs and other soluble factors, including MUC-2, antimicrobial peptides, and anti-inflammatory cytokines (7, 28, 29). However, nothing is known about the effects of BFT-2-containing OMVs secreted by *Bacteroides* on primary human colon organoids.

In contrast to ETBF, NTBF does not express *bft-2*. NTBF promotes gut homeostasis through the production of short-chain fatty acids and polysaccharide A, stimulates barrier-protective molecules including ZO-1 and MUC-2, and promotes the secretion of antimicrobial peptides and anti-inflammatory cytokines (1, 7, 30).

A recent review questioned the relevance of cell lines as models to elucidate the physiology and pathophysiology of the colon since cell lines are rarely normal and contain several mutations and chromosomal defects that enable their persistence (31). In particular, it has been reported that the gene expression pattern of the Caco-2 cell line, which is studied as a surrogate of the colon, was similar to that of epithelial cells of the small intestine (32). Since, as noted above, colon cell lines do not produce consistent responses to recombinant BFT-2 (3, 9, 10), one of the most important challenges involves the assessment of BFT-2 secreted in OMVs on primary human colon organoids (1). To elucidate the biological activity of BFT-2 secreted in OMVs, we obtained colon biopsies from multiple healthy human donors to develop differentiated two-dimensional monolayers of primary colon organoids. Primary colon organoids contain multiple cell populations found in native tissue, compared to cell lines, which do not fully differentiate (31). Accordingly, the goals of this study were to develop a model to study host-pathogen interactions in primary human colon organoids and to test the hypothesis that BFT-2 secreted in OMVs modulates mucosal immunity, TER, and CFTR Cl⁻ secretion, a major driver of fluid secretion in the colon (14). Notably, it has been demonstrated that BFT is not a freely secreted protease but is associated with OMVs (12). We report that OMVs had no effect on cell adhesion or tight junction proteins, notably E-cadherin and claudin-2, TER, or sodium reabsorption, but did inhibit forskolin-stimulated CFTR Cl⁻ secretion, an effect predicted to decrease fluid secretion by the colon. NTBF OMVs containing BFT-2 had a more inhibitory effect on CFTR Cl⁻ secretion by primary human colonoids than NTBF OMVs lacking BFT-2. By contrast, deletion of *bft-2* in ETBF had no significant effect on CFTR Cl⁻ secretion by colon organoids compared to ETBF. In addition, OMVs were not pro-inflammatory as determined by measuring the secretion of 48 cytokines. OMVs also reduced CFTR Cl⁻ secretion by T84 cells, a colon cell line, but did not alter TER. These results demonstrate that OMVs secreted by *B. fragilis* have very different biological effects on primary human colon organoids compared to colon cell lines exposed to recombinant BFT-2.

## RESULTS

### Isolation and characterization of OMVs secreted by *Bacteroides*

**Fig 1:**
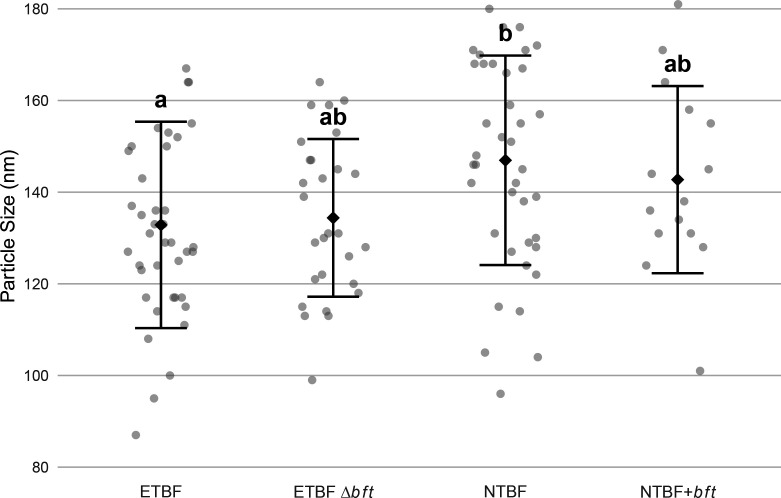
*Bacteroides* OMV particle size determined by NTA. Each gray circle represents a single NTA measurement. The number of measurements per strain ranged from 15 to 35. Group means (black diamonds) and error bars (±SD) are also presented. Letters above bars indicate statistical groupings based on one-way ANOVA with Tukey’s HSD post-hoc test. Experimental groups sharing the same letter are not significantly different from each other (*P* > 0.05), while groups with different letters are significantly different from each other (*P* < 0.05).

To test the hypothesis that BFT-2 in OMVs leads to a disruption in the integrity of epithelial barriers and modulates CFTR Cl⁻ secretion, we utilized four strains of *B. fragilis*: ETBF and ETBF in which the *bft-2* gene was deleted (ETBF Δ*bft*); NTBF, a strain that does not express *bft-2*; and NTBF in which the *bft*-2 gene was expressed (NTBF+*bf*t). Nanoparticle tracking analysis (NTA), which measures the size of vesicles by dynamic light scattering, revealed that the size of OMVs isolated from bacterial cultures was similar for most strains, except that ETBF OMVs were significantly smaller than NTBF OMVs (Fig. 1).

**Fig 2:**
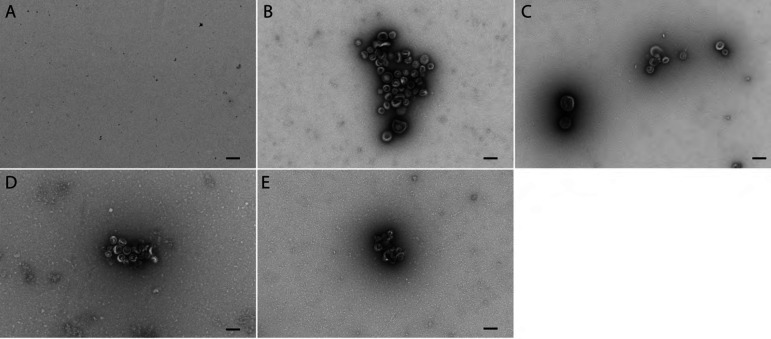
Representative transmission electron microscopy images of negatively stained *Bacteroides* OMVs. (**A**) Process control. (**B**) ETBF. (**C**) ETBF Δ*bft*. (**D**) NTBF. (**E**) NTBF+*bft*. Images taken by an observer blinded to the origin of the OMVs. Scale bars: 100 nm.

According to NTA analysis, the number of OMVs in each bacterial culture was 1.4 × 10^11^ particles/mL for ETBF, 2.8 × 10^11^ particles/mL for ETBF Δ*bft*, 2.0 × 10^11^ particles/mL for NTBF, and 2.2 × 10^11^ particles/mL for NTBF+*bft*. To confirm that we isolated OMVs, transmission electron microscopy (TEM) was utilized to image OMVs (Fig. 2). OMVs were not observed in the process control (PC, bacterial culture media not exposed to bacteria and run through the OMV isolation process); however, OMVs were observed in media isolated from ETBF, NTBF, ETBF Δ*bft,* and NTBF+*bft* (Fig. 2). The mean diameter (±SEM) of OMVs, as measured by TEM, was similar in all groups (*P* > 0.77): ETBF (59.0 nm ± 2.0 nm, *n* = 31), ETBF Δ*bft* (63.2 ± 3.6 nm, *n* = 21), NTBF (60.2 ± 4.0 nm, *n* = 14), and NTBF+*bft* (58.0 ± 5.0 nm, *n* = 11). Most *Bacteroides* OMVs had typical morphology, but we also observed some collapsed vesicular forms and irregularly shaped vesicles, similar to images published recently for *Bacteroides* OMVs secreted by ETBF and NTBF (33). As expected, the size of OMVs was assay-dependent: dynamic light scattering tends to overestimate size, whereas TEM tends to underestimate size (34, 35).

**Fig 3:**
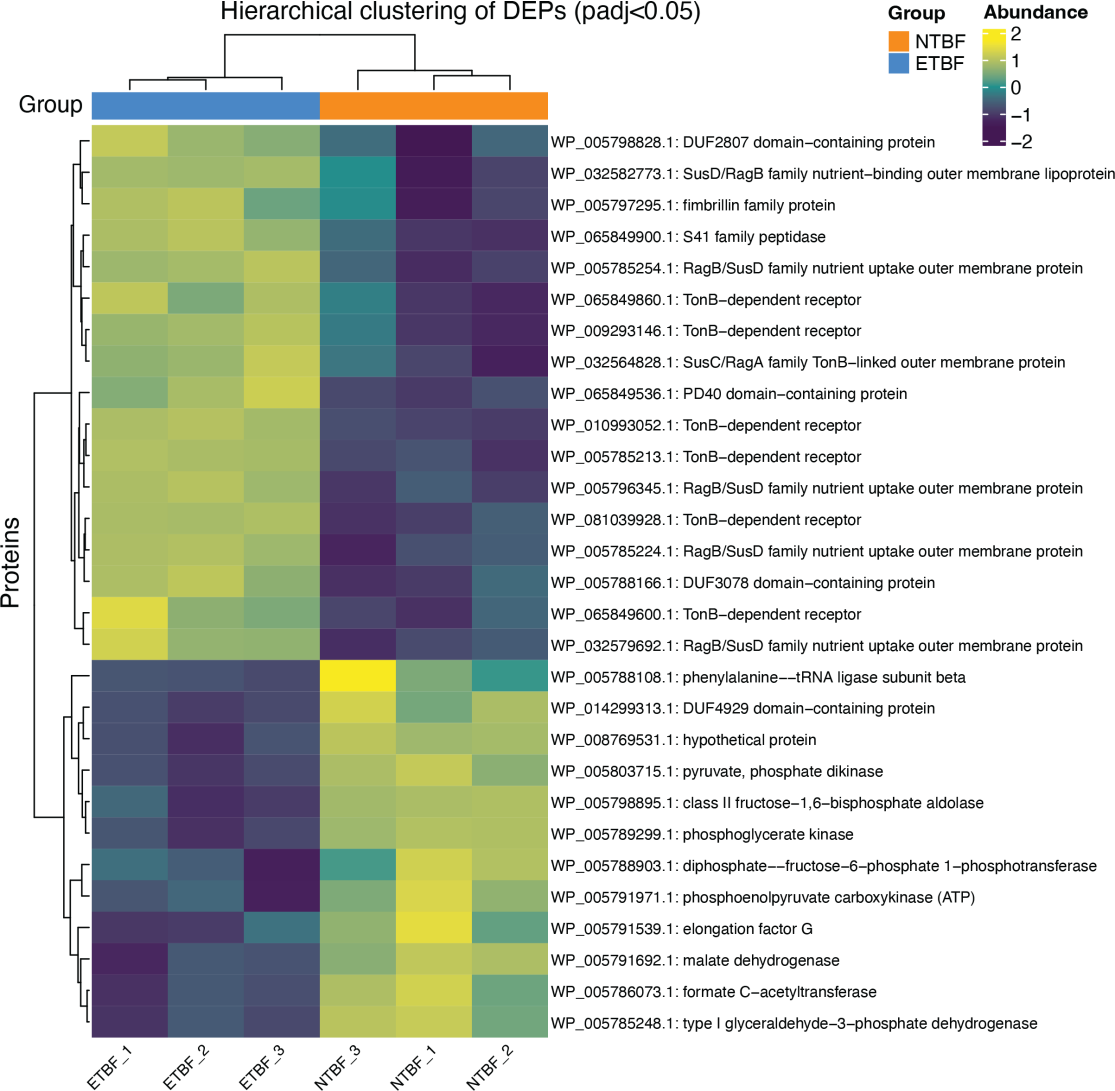
ETBF and NTBF OMV proteomes. Differential abundance analysis reveals significant differences between the proteome of ETBF and NTBF OMVs. Heatmap showing differentially expressed proteins in ETBF OMVs and NTBF OMVs with adjusted *P*-values < 0.05. Strains and replicate numbers are labeled for each column, and protein accession numbers and descriptions are labeled for each row. Both rows and columns are clustered according to Euclidean distance measurements. NTBF (orange) and ETBF (blue) replicates are colored at the top of each column. Abundance measurements are scaled on a per row basis. Abundance is presented as log2 iBAQ (intensity-based absolute quantification). The list of all identified proteins by proteomics analysis is presented in the Supplemental Tables. Table S1 includes proteins detected exclusively in NTBF OMVs. Table S2 includes proteins detected exclusively in ETBF OMVs. Table S3 includes proteins detected in both ETBF and NTBF OMVs. Finally, Table S4 includes differential expression of proteins in ETBF and NTBF OMVs, the results of which are depicted in this figure. In accordance with previous research, our results show that several TonB-dependent receptors are more abundant in OMVs secreted by ETBF compared to NTBF strains (37).

Analysis of the proteome of OMVs was conducted, in part, to confirm that BFT-2 was present in ETBF and NTBF+*bft* and absent in ETBF Δ*bft* and NTBF. Results of the proteomics analysis of OMVs are presented in Fig. 3; Tables S1 to S4. We detected 568 proteins in NTBF OMVs and 437 proteins in ETBF OMVs, with 247 proteins being found in both strains. BFT was detected in all three biological replicates of ETBF, although the associated protein fragipain was not detected in any of the replicates. Fragipain can cleave BFT-2 into its active form, but studies have shown that it is not required to activate BFT (36). TonB-dependent receptors were significantly more abundant in ETBF compared to NTBF strains. TonB-dependent receptors are one part of outer membrane structures in *B. fragilis* that are critical for the influx of nutrients via SusC and SusD, both outer membrane proteins (37), which were also increased in ETBF compared to NTBF OMVs. An increase in the abundance of these proteins may suggest an enhanced need for ETBF to improve nutrient acquisition in the microbiota, thereby enhancing its ability to compete for niches that NTBF can more easily occupy.

Using an intensity-based quantification approach to measure BFT-2 in OMVs, the amount of BFT-2 was similar in ETBF OMVs and NTBF+*bft* OMVs (log_2_ 12.43 for NTBF+*bft* and log_2_ 12.90 for ETBF). BFT-2 could not be detected by intensity-based quantification in NTBF and ETBFΔ*bft* OMVs. Thus, two complementary proteomic approaches confirmed the presence of BFT-2 in OMVs secreted by ETBF and NTBF+*bft*, but not in OMVs secreted by NTBF and ETBF.

### Characterization of primary human colon organoids: development of a new model to study host-pathogen interactions in the colon

**Fig 4:**
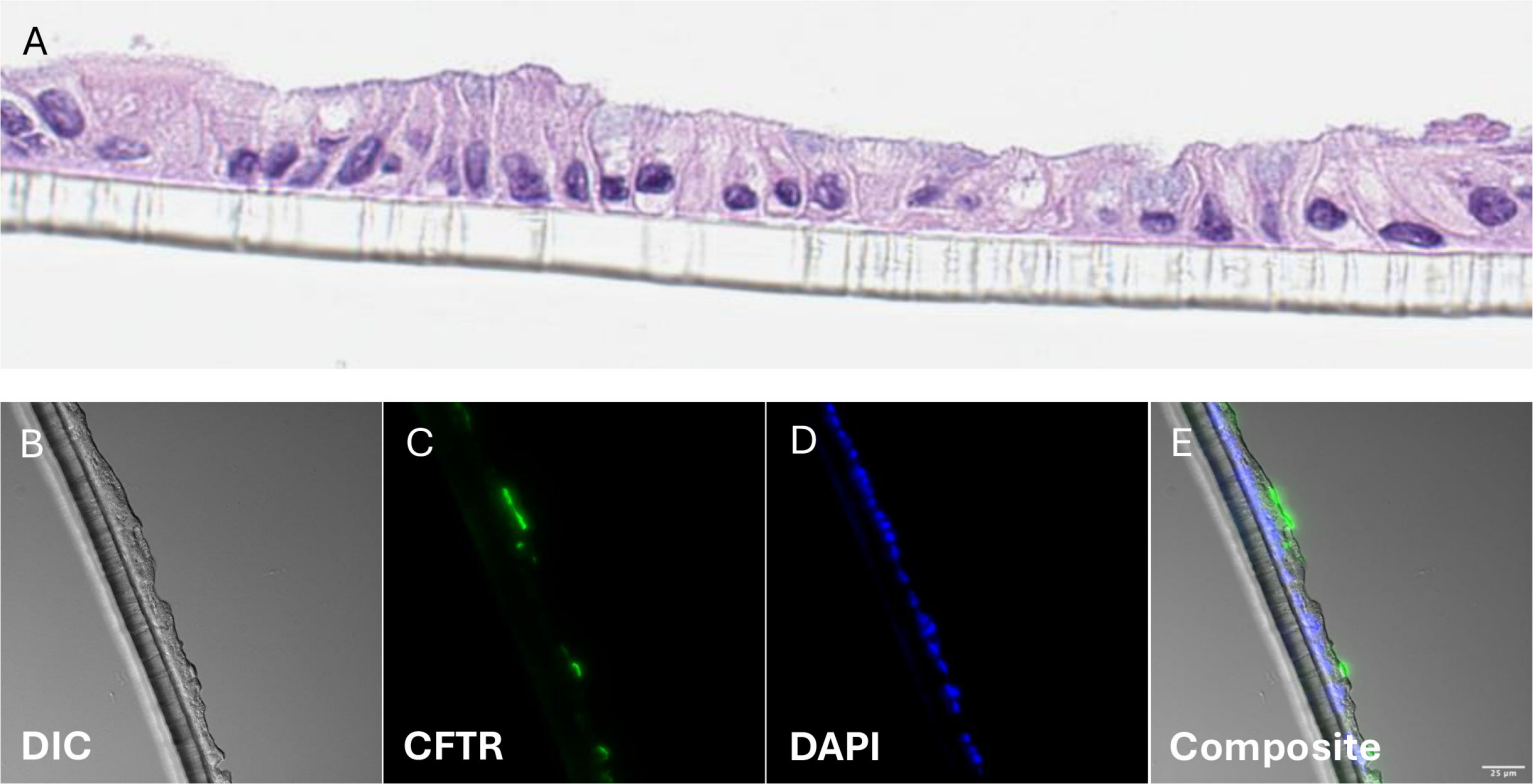
Representative images of human colon organoids. (**A**) Cross section of a confluent monolayer of colon organoids grown on Transwell filters demonstrating an intact and heterogeneous epithelium (10 μm thick: H&E-stained cells). (**B**) Differential contrast (DIC) image of a 6 μm thick frozen cross-section of differentiated colon organoids on Transwell filters. (**C**) Immunofluorescent image of CFTR in the same colon organoid as in panel **B**. (**D**) DAPI nuclear labeling of the same colon organoid monolayer as in panels **B** and **C**. (**E**) Composite of panels **B–D** showing apical membrane labeling of CFTR. Scale bar in panel **E** is 50 μm.

**Fig 5:**
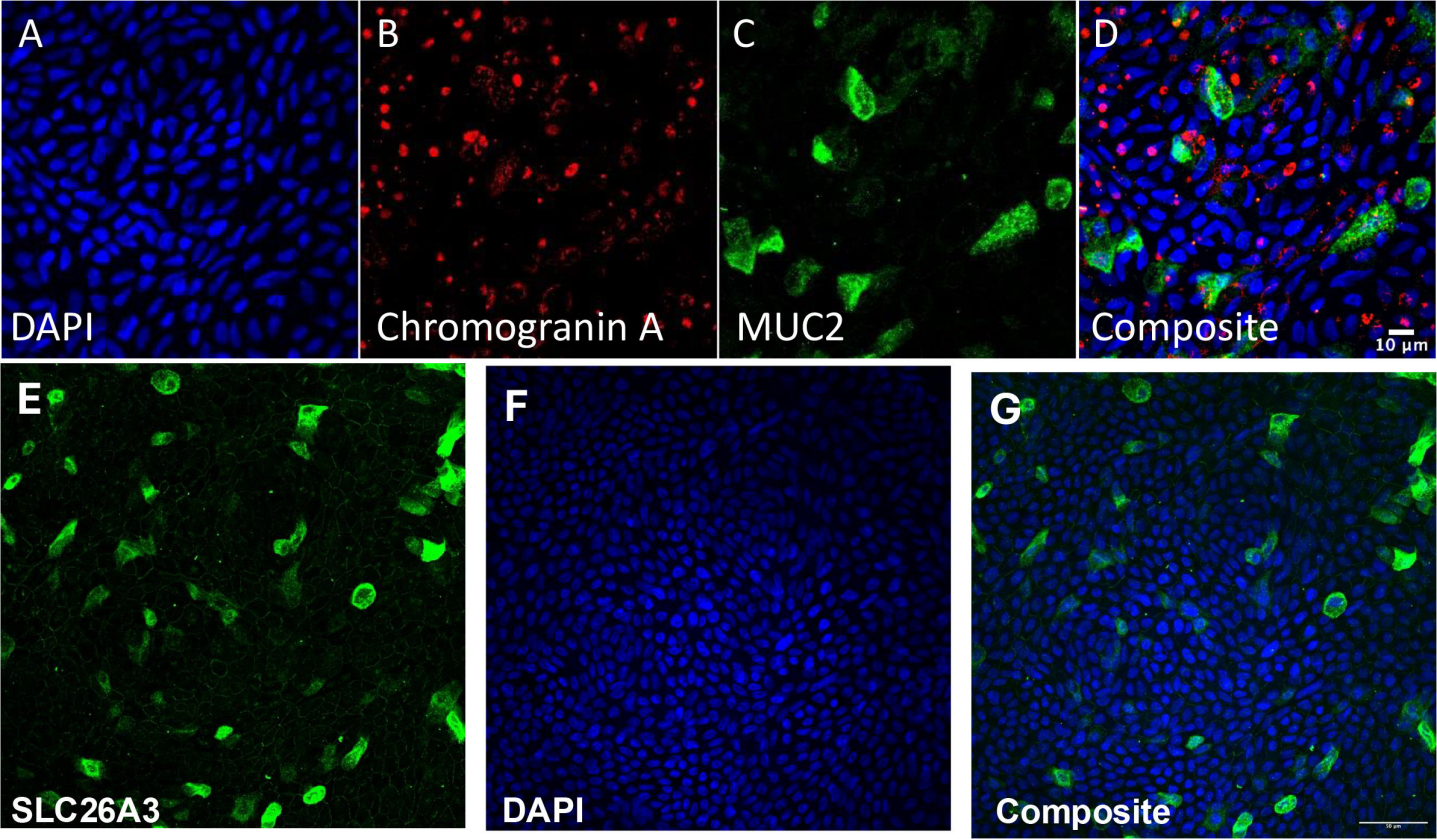
Representative *en face* images of human colon organoids on Transwell filters. (**A**) DAPI labeling of nuclei. (**B**) Chromogranin A immunofluorescence of enterochromaffin cells. (**C**) Immunofluorescence of MUC2. (**D**) Composite of panels A–C revealing that MUC2 and Chromogranin A are expressed in different cell types. (**E**) Immunofluorescence of SLC26A3 (DRA, a Cl⁻/HCO⁻_3_ exchanger). (**F**) DAPI labeling of nuclei. (**G**) Composite of panels E and F. Scale bar in panel g is 50 μm

Biopsies of the human proximal colon were obtained during clinical colonoscopy after written informed consent was obtained from healthy volunteers at Dartmouth Health. As described in the “Materials and Methods” section, crypt cells were dispersed and grown in Matrigel until 3D colon organoids were formed. The 3D colon organoids were then seeded on Transwell filters and grown for one week to develop polarized, differentiated colon organoids. We used an imaging approach to characterize the cellular composition of colon organoids *in vitro* as monolayers. Figure 4 and 5 reveal that the colon organoids contain cell types found in the proximal colon, including mucin (MUC2)-producing cells, CFTR-expressing cells, SLC26A3-expressing cells, and enterochromaffin cells (14, 38). These observations indicate that the human primary colon organoids form a well-differentiated epithelium, contain cell types similar to the colon *in vivo*, and provide a new model to study host-pathogen interactions (14, 38).

### **OMVs inhibit CFTR Cl**⁻ **secretion by primary human colon organoids**

**Fig 6:**
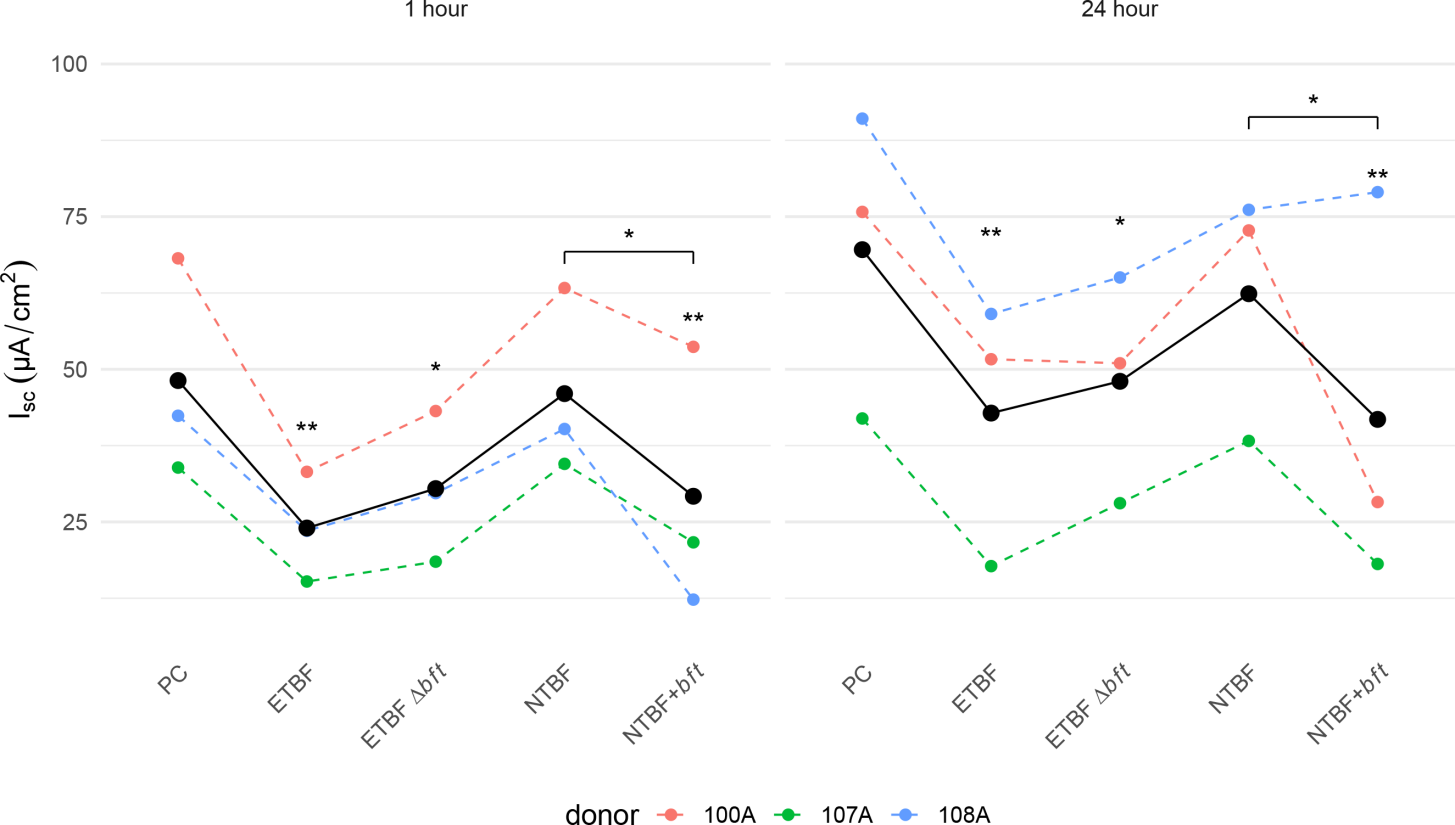
CFTR Cl⁻ secretion by human primary colon organoids measured as short-circuit current (Isc, μA/cm^2^). Left: 1 h after exposure to PC or OMVs (2 × 10^10^/mL OMVs, a concentration of OMVs similar to that measured in conditioned media and in biological fluids (21, 40). Right: 24 h after a 1 h exposure to PC or OMVs (2 × 10^10^/mL. Dashed colored lines connect data from the same donor across treatment conditions, illustrating the within-donor paired structure of the data that is accounted for by the mixed-effects model (*n* = 3 donors; Donor 108A is female, Donors 100A and 107A are male). The black circles represent the mean currents. Amiloride (50 μM) was added first to the apical side of cells to assess sodium reabsorption, followed by forskolin (10 μM), and then CFTR_inh_-172 (20 μM), which inhibits forskolin-stimulated CFTR Cl⁻ secretion. CFTR Cl⁻ secretion is reported as the CFTR_inh_-172 inhibited forskolin-stimulated current. Many significant differences from PC were observed at 1 h (ETBF, **P = 0.0021; ETBF Δ*bft*, *P = 0.0113; and NTBF+*bft*, **P = 0.0080). Expression of *bft* in NTBF (NTBF+*bft*) OMVs reduced CFTR Cl⁻ secretion at 1 h compared to NTBF OMVs, as indicated by the horizontal bar (*P = 0.0144). At 24 h, many significant differences from PC were observed (ETBF **P = 0.0098; ETBF Δ*bft;* *P = 0.0265, and NTBF+*bft* **P = 0.0081). The horizontal bar indicates that NTBF+*bft* OMVs significantly reduced CFTR Cl⁻ secretion (*P = 0.0320) compared to NTBF OMVs. Statistical analysis was performed using mixed-effects linear models with donor as a random effect for each time point separately. Experiments were performed with three donors of colon cells, three technical replicates per donor, and three different preparations of OMVs. Three technical replicates were averaged as a single data point.

**Fig 7:**
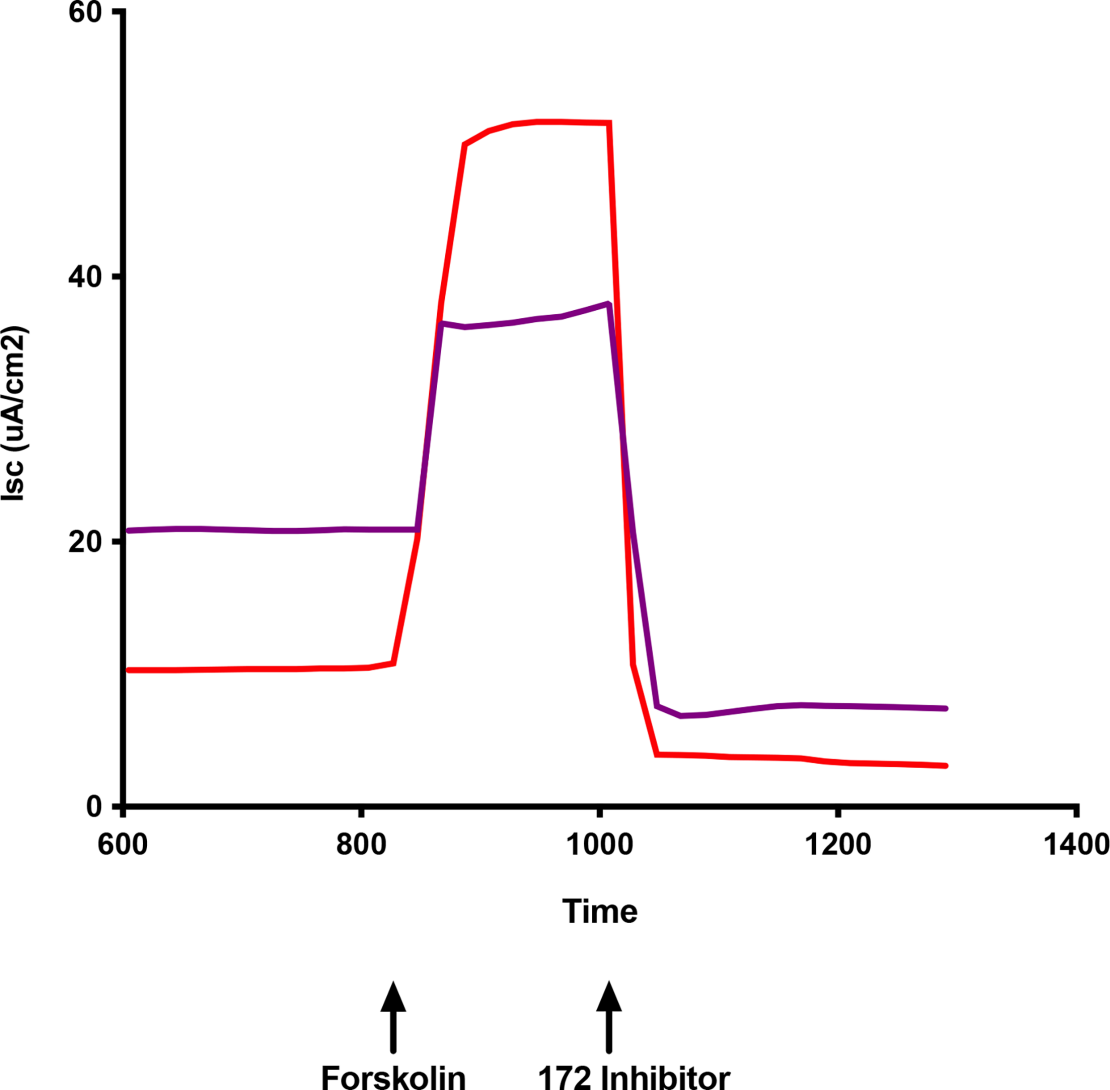
Representative current traces of CFTR Cl⁻ secretion by human primary colon organoids measured as short-circuit current (Isc) in Ussing chambers. After ~10 min for the current to reach a steady-state, forskolin (10 μM) was added to the apical solution, followed by CFTR_inh_-172 (20 μM), which inhibits forskolin-stimulated CFTR Cl⁻ secretion. PC, red trace. ETBF, purple trace.

Previous studies have shown that recombinant BFT-2 stimulates CFTR Cl⁻ secretion when added to the basolateral side of T84 cells (20). To examine the effect of OMVs secreted by *B. fragilis* on CFTR Cl⁻ secretion by primary human colon organoids, OMVs (2 × 10^10^/mL) were isolated and applied to the apical side (the side of the colon exposed to *Bacteroides in vivo*) of colon organoids for 1 h, and then forskolin-stimulated CFTR Cl⁻ secretion was measured in Ussing chambers immediately after the 1 h exposure or 24 h after washing the OMVs from the organoids. Forskolin increases the activity of protein kinase A, which phosphorylates and activates CFTR Cl⁻ secretion (39). Figure 6 demonstrates that OMVs secreted by ETBF, ETBF Δ*bft,* and NTBF+*bft* reduced forskolin-stimulated CFTR Cl⁻ secretion after 1 h of exposure compared to process control (PC). Notably, OMVs secreted by ETBF reduced Cl⁻ secretion significantly more than OMVs secreted by NTBF, which do not express BFT-2. Expression of *bft* in NTBF (NTBF+*bft*) reduced CFTR Cl⁻ secretion compared to NTBF OMVs, which do not express BFT-2. By contrast, OMVs isolated from ETBF Δ*bft* had no effect on CFTR Cl⁻ secretion compared to ETBF OMVs containing BFT-2. This observation suggests that the ability of BFT-2 to inhibit CFTR Cl⁻ secretion is context-dependent (i.e., strain). Figure 7 presents representative current traces of CFTR Cl⁻ secretion by colon organoids exposed to PC and ETBF for 1 h.

Twenty-four hours after a 1 h exposure to OMVs, CFTR Cl⁻ secretion was not significantly reduced by ETBF, ETBF Δ*bft*, or NTBF when compared to PC (Fig. 6). However, 24 h after exposure to NTBF+*bft* OMVs, CFTR Cl⁻ secretion was less than CFTR Cl⁻ secretion compared to NTBF OMVs and PC (Fig. 6). This observation suggests that the ability of BFT-2 in OMVs to inhibit CFTR Cl⁻ secretion is strain dependent.

**Fig 8:**
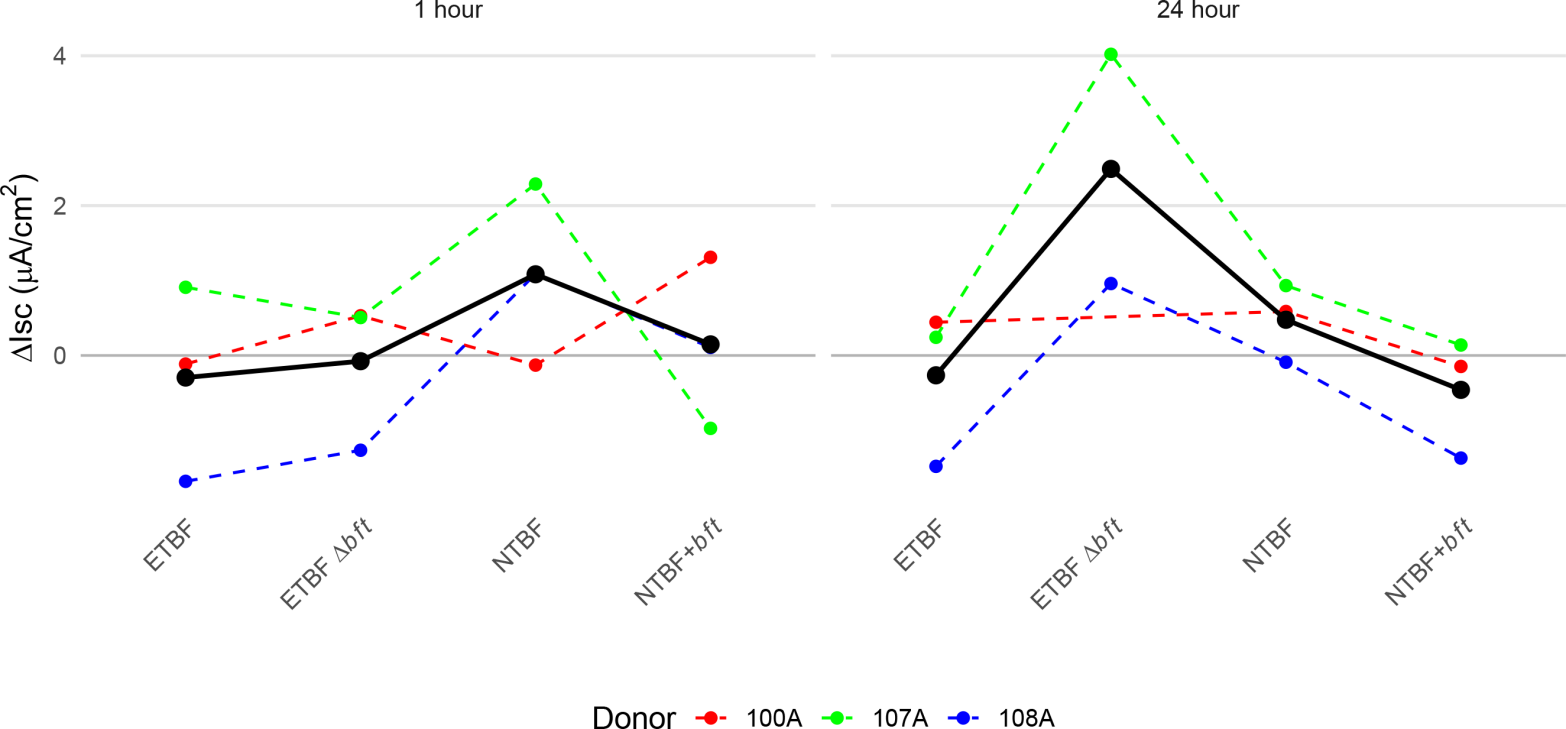
OMVs did not modify amiloride-sensitive short-circuit Na^+^ currents. Amiloride-sensitive Na^+^ currents in response to OMVs (2 × 10^10^/mL OMVs). The difference in amiloride-sensitive currents (ΔIsc) between PC and OMV-exposed colon organoids is plotted. Colon organoids were exposed to OMVs for 1 h, and ΔIsc was measured immediately after the 1 h exposure or 24 h after the 1 h exposure to examine the longer-term effect. Each measurement of amiloride-sensitive current for each donor was adjusted by subtracting the process control (PC) current from the same Ussing chamber session and the same donor. Data from each donor (100A = red, 107A = green, 108A = blue) are depicted with dashed lines. The back circles represent the mean currents. The horizontal line at zero represents no change from PC. Statistical analysis was performed using one-sample *t*-tests of the PC-adjusted current (ΔIsc) against zero for each time point separately, with technical replicates averaged within donor prior to analysis. No significant differences from PC were observed for any treatment at either time point (*P* > 0.05, *n* = 3 donors, except ETBF Δbft at 24 h, where *n* = 2).

OMVs had no effect on the amiloride-sensitive short-circuit Na^+^ currents, either after a 1 h exposure to OMVs or after 24 h following the 1 h exposure to OMVs (Fig. 8).

Experiments were conducted using colon cells from three donors, with three technical replicates per donor, and three different preparations of OMVs. Three technical replicates were averaged as a single data point.

**Fig 9:**
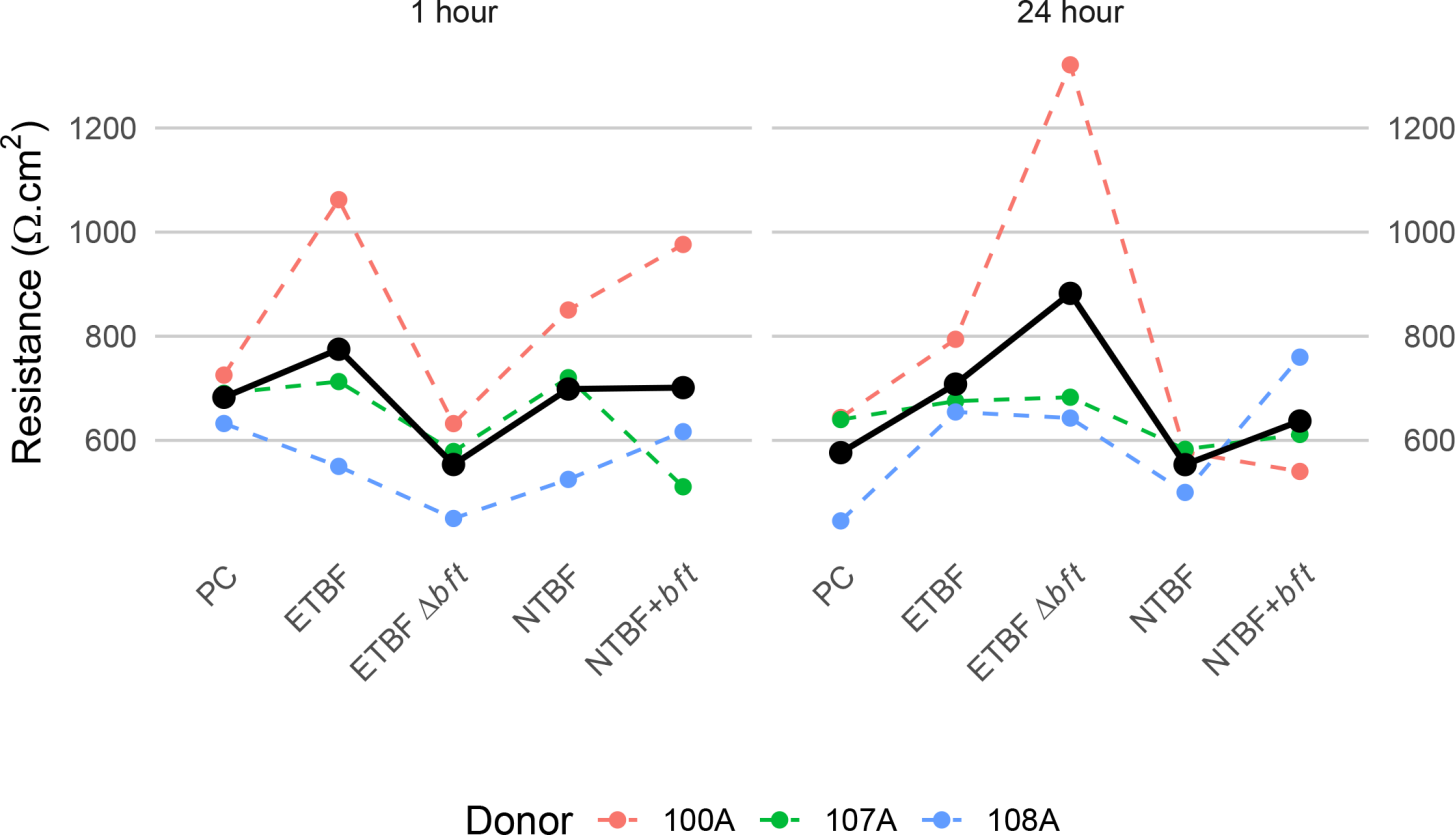
Transepithelial resistance (TER) of primary human colon organoids. TER was measured in colon organoids in which CFTR Cl⁻ secretion was measured 1 h (left figure) or 24 h (right figure) after exposure to PC or OMVs (2 × 10^10^/mL) secreted by ETBF, ETBF Δ*bft*, NTBF, and NTBF+*bft*. Individual donor responses are shown as dashed colored lines, with each color representing a different donor. Statistical analysis was performed using a mixed-effects linear model with donor as a random effect and hour as a fixed covariate. Experiments were performed with three donors of colon cells, three technical replicates per donor, and three different preparations of OMVs. Three technical replicates were averaged as a single data point. No significant differences compared to PC (1 h and 24 h exposure) were observed (*P* > 0.05).

Experiments were also conducted to determine if OMVs had an effect on TER, since previous studies have shown that recombinant BFT-2 reduced TER in several colon cell lines (9, 15, 17, 19, 20). However, none of the OMVs had a significant effect on TER (Fig. 9) compared to process control (PC) at 1 h and 24 h after the 1 h exposure to OMVs.

### *Bacteroides* OMVs are not cytotoxic to colon organoids

**Fig 10:**
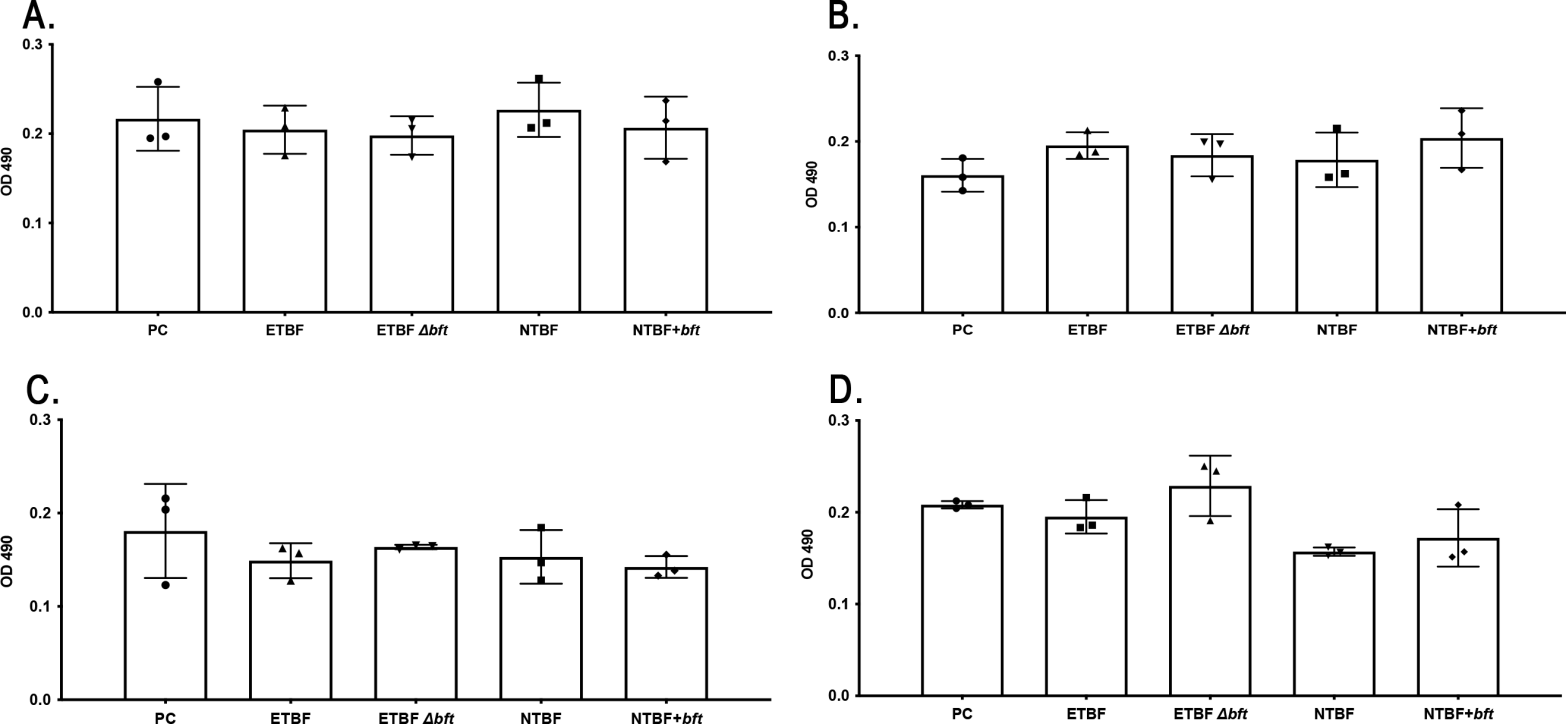
OMVs are not cytotoxic to colon organoids. LDH release by colon organoids. (**A**) Apical LDH 1 h after OMV exposure. (**B**) Basolateral LDH 1 h after OMV exposure. (**C**) Apical LDH 24 h after OMV exposure. (**D**) Basolateral LDH 24 h after OMV exposure. LDH release from three donors of colon cells, three technical replicates of OMVs per donor, and three different preparations of OMVs. Three technical replicates were averaged as a single data point for each donor. No significant differences compared to PC (1 h and 24 h exposure) were observed as determined by a one-way ANOVA (*P* > 0.05).

To determine if OMVs were cytotoxic to colon organoids, LDH was measured in conditioned cell culture media obtained from apical and the basolateral compartment at the end of the 1 h exposure and 24 h after exposure to OMVs. OMVs did not have a significant effect on LDH release compared to PC exposure in any experimental group (Fig. 10).

### *Bacteroides* reduces CFTR Cl⁻ secretion by T84 cells but has no effect on TER

**Fig 11:**
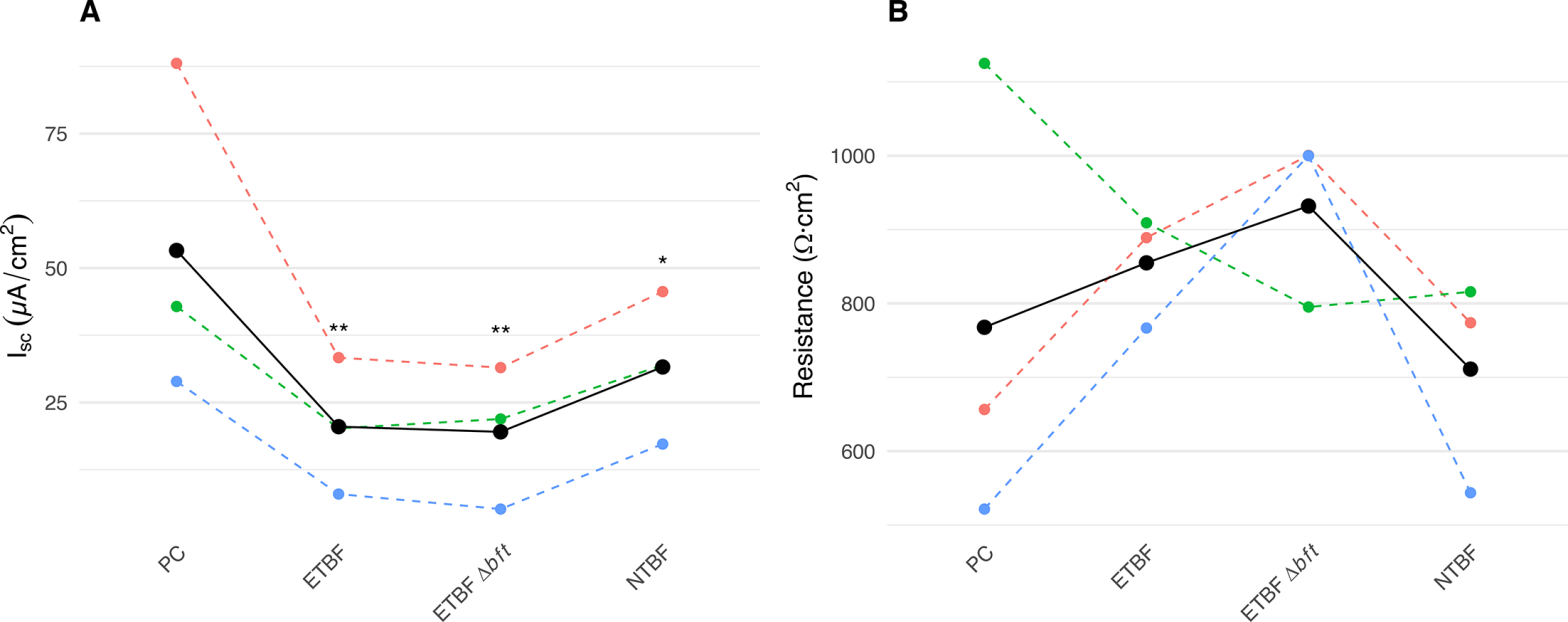
CFTR Cl⁻ secretion and TER by T84 cells exposed to OMVs. The protocol was identical to that performed on primary human colon organoids. T84 monolayers were exposed to OMVs (2 × 10^10^/mL) for 1 h, and CFTR Cl⁻ secretion (short-circuit current: Isc) was measured. Amiloride (50 μM) was added first to the apical side of cells to assess sodium reabsorption, followed by forskolin (10 μM), and then CFTR_inh_-172 (20 μM), which inhibits forskolin-stimulated CFTR Cl⁻ secretion. CFTR Cl⁻ secretion is reported as the CFTR_inh_-172 inhibited forskolin-stimulated current. Data represent individual experiments (dashed lines) grouped by experiments performed on the same day on the same pass of cells (colors). (**A**) CFTR Cl⁻ secretion. Each line connects experiments conducted on the same day on the same pass of T84 cells. Compared to PC, CFTR Cl⁻ secretion was significantly reduced by ETBF (**P = 0.006), ETBF Δ*bft* (**P = 0.005), and NTBF (*P = 0.032) OMVs. (**B**) TER in the same set of T84 cells used for measurements of CFTR Cl⁻ secretion. No significant differences in TER compared to PC were observed after 1 h of exposure to OMVs. Statistical analysis was performed using mixed-effects linear models with donor as a random effect for each time point separately. Experiments were performed with three different preparations of OMVs. Three technical replicates were averaged as a single data point.

We also conducted studies on T84 cells, a colon cell line that has been used to study the effects of recombinant BFT-2 (41–43). Previous studies have shown that recombinant BFT-2 reduces the TER in several colon cell lines (9, 15, 17, 19, 20). To determine if *B. fragilis* OMVs also reduced TER and influenced CFTR Cl⁻ secretion, we conducted Ussing experiments on monolayers of T84 cells under conditions identical to the previously described exposure of primary human colon organoids. Compared to PC, CFTR Cl⁻ secretion was reduced by all OMVs (Fig. 11A). ETBF OMVs were not more effective than NTBF OMVs in reducing CFTR Cl⁻ secretion. Moreover, deletion of *bft-2* in ETBF did not significantly reduce the effect of ETBF OMVs on CFTR Cl⁻ secretion. OMVs also had no effect on TER (Fig. 11B). Thus, OMVs reduced CFTR Cl⁻ secretion by both primary human colon organoids and T84 cells, without altering TER.

### OMVs do not alter tight junction or cell adhesion protein abundance

**Fig 12:**
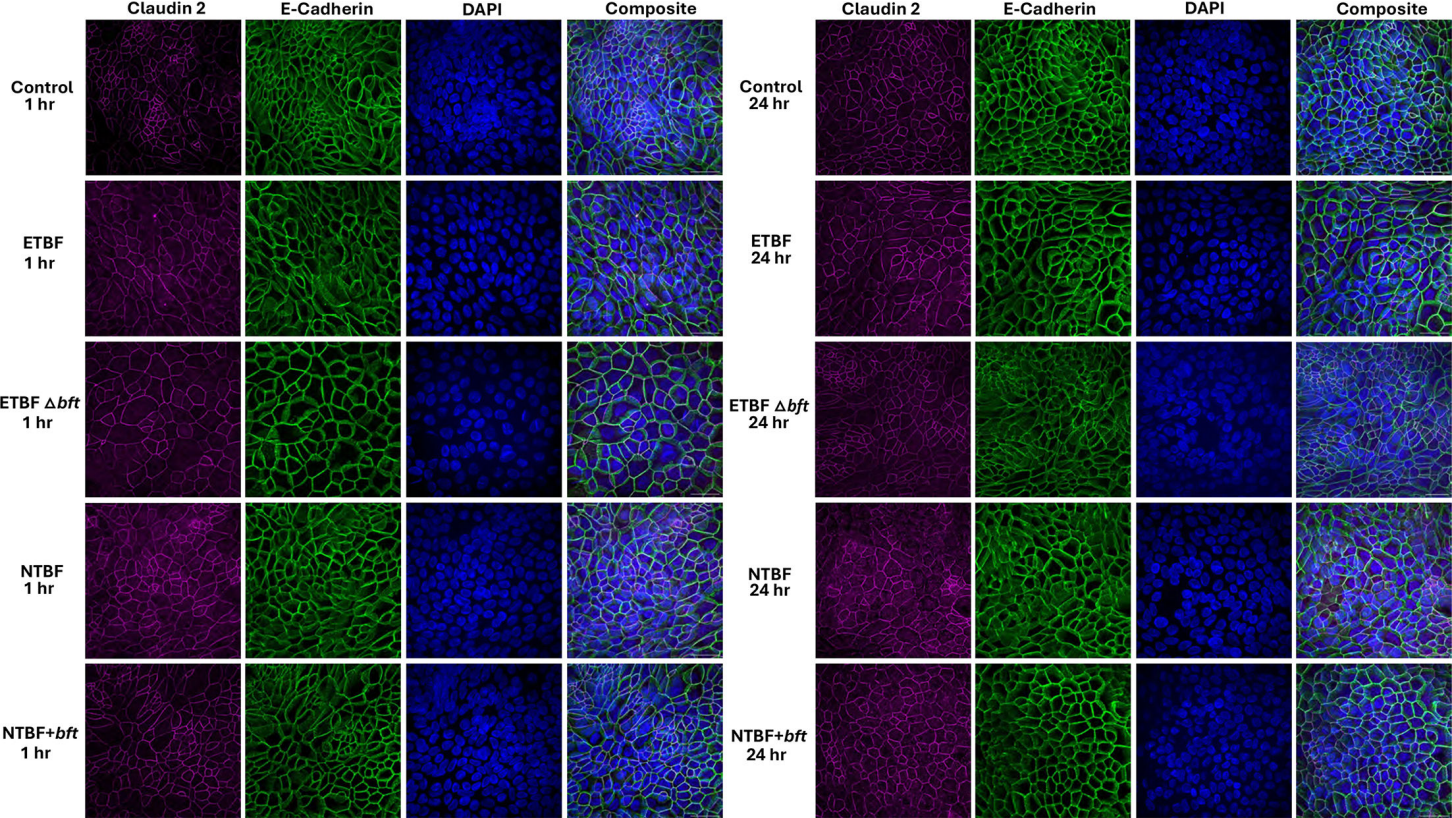
Representative confocal images of human colon organoids. Organoids were grown on Transwell filters and exposed to OMVs (2 × 10^10^/mL) for 1 h. Claudin-2 (magenta) and E-cadherin (green) were identified by immunofluorescence. DAPI (blue) identified nuclei. Confocal images were analyzed by an investigator who was blinded to the treatment. There was no difference in claudin-2 or E-cadherin abundance using confocal microscopy and the Fiji open-source image processing package to quantify fluorescence intensity (see Fig. 13 for data).

Previous studies have also shown that recombinant BFT-2 rapidly (<1 h) reduced the expression of the cell adhesion protein E-cadherin in cell lines (9, 15, 17, 19, 20). However, we observed no effect of *Bacteroides* OMVs on the abundance of E-cadherin or on the tight junction protein claudin-2 in human colon organoids after 1 h and 24 h, following the 1 h exposure to OMVs (2 × 10^10^/mL), as determined by immunofluorescence microscopy (Fig. 12).

**Fig 13:**
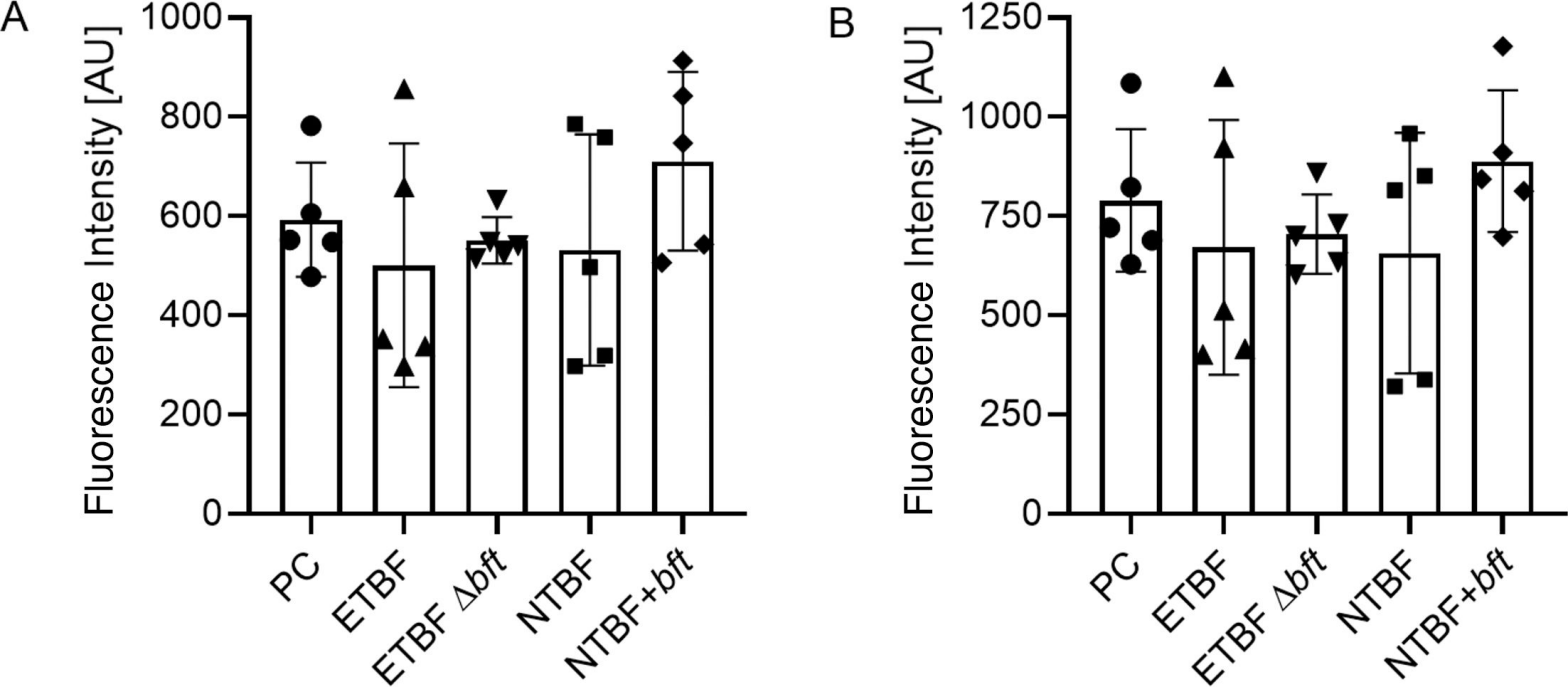
Measurements of E-cadherin (**A**) and claudin-2 (**B**) epifluorescence. Data were generated by a co-author blinded to the experimental treatment. Five random images were chosen for each treatment. There was no significant effect of OMVs (2 × 10^10^/mL) on E-cadherin or claudin-2 abundance. AU, arbitrary fluorescent units. Data are expressed as mean ± one standard deviation.

Studies were also conducted to measure E-cadherin and claudin-2 fluorescence by confocal microscopy. None of the OMVs had a significant effect on E-cadherin or claudin-2 fluorescence (Fig. 13), confirming the immunofluorescent results shown in Fig. 12.

### Cytokine secretion by human primary colon organoids

**Fig 14:**
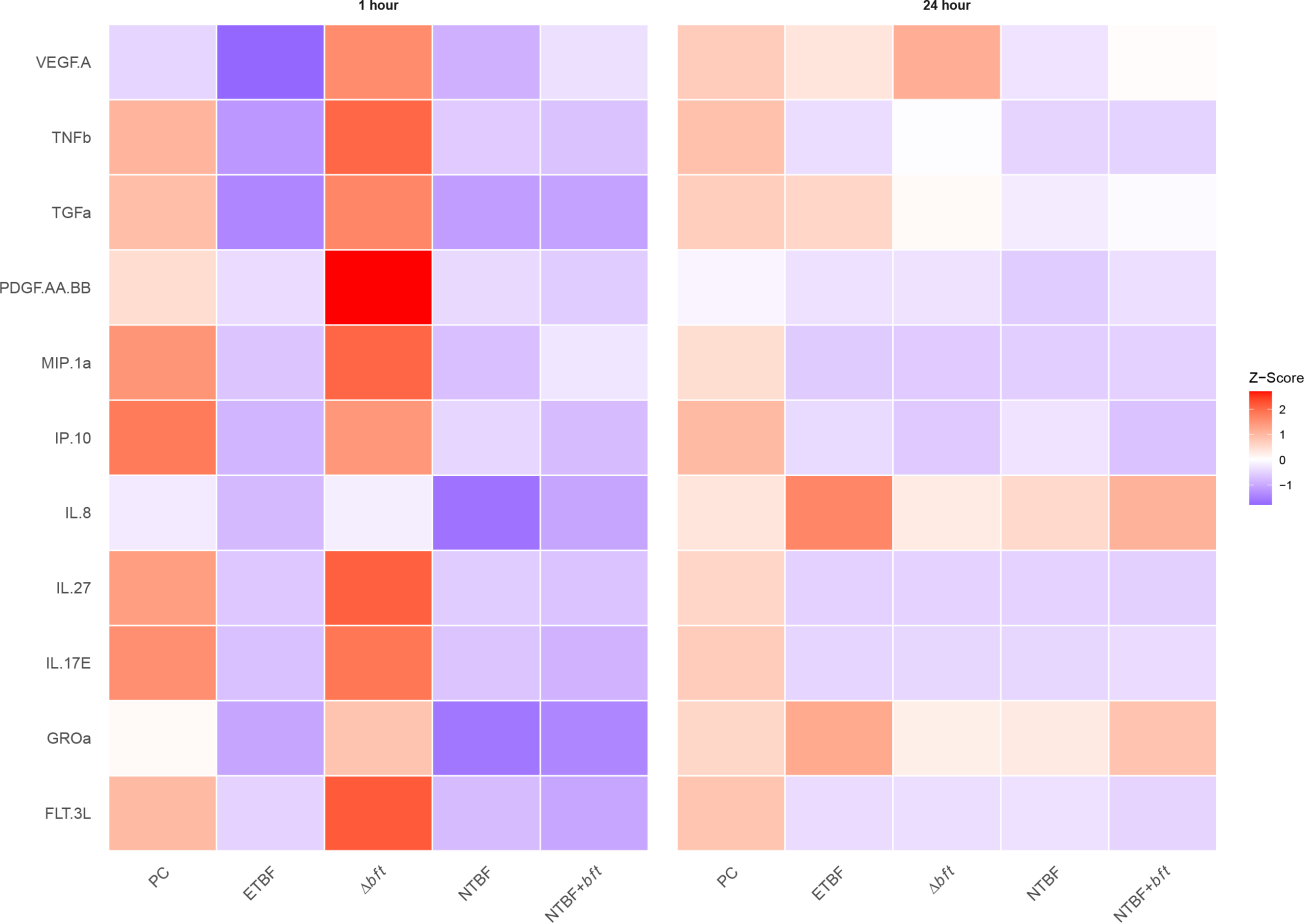
Cytokine secretion following 1 h and 24 h after exposure to OMVs secreted by *Bacteroides fragilis* or to PC. There were no significant effects of OMVs (2 × 10^10^/mL) versus PC. Values represent means across all donors (*n* = 3 donors) for each treatment condition. Color scale indicates standard deviations from the time point mean: red represents above-average expression, blue represents below-average expression, and white represents average expression for each cytokine at each time point. Only cytokines with complete data (values above detection) across all conditions are shown. Data were log2-transformed prior to z-score calculation.

Previous studies on cell lines and human colon tissue reported that *B. fragilis* and recombinant BFT-2 stimulate IL-8 secretion (9, 15, 17–20). To examine the effect of OMVs on the innate immune response, colon organoids were grown on Snapwell filters and exposed to PC or OMVs (2 × 10^10^/mL) in the apical media for 1 h or 24 h after OMVs were removed by washing. Fig. 14 demonstrates that OMVs had no significant effect on IL-8 secretion compared to PC. Moreover, there was no significant effect of any OMVs compared to PC on the secretion of 48 cytokines measured using the MILLIPLEX MAP Human Cytokine/Chemokine 48-Plex cytokine assay, although there were some trends in the response. For example, compared to PC, 24 after exposure to ETBF OMVs and NTBF+*bf*t OMVs, IL-8 increased compared to PC; however, the changes were not statistically significant.

## DISCUSSION

There were two primary goals of this study: (1) to develop a well-differentiated primary culture of human colon organoids and (2) to elucidate the effect of endogenous BFT-2 in OMVs secreted by *B. fragilis* on human primary colon organoids. We successfully developed a model of colon organoids from three donors that displayed a differentiated epithelium containing cell types found in the proximal colon, including mucin (MUC2)-producing cells, CFTR-expressing cells, SLC26A3 (DRA, a Cl⁻/HCO⁻_3_ exchanger that plays a key role in fluid absorption and enterocyte acid/base balance)-expressing cells, and enterochromaffin cells, which underlie epithelial cells and secrete serotonin to stimulate GI motility and fluid and electrolyte secretion (14, 38). These observations indicate that the human primary colon organoids are a representative model to study host-pathogen interactions in the human colon (14, 38). Studies were also conducted to examine the effect of OMVs on CFTR Cl⁻ secretion, amiloride-sensitive sodium absorption, TER, and cytokine secretion. We found that OMVs secreted by most strains of *Bacteroides*, except NTBF, significantly reduced CFTR Cl⁻ secretion by colon organoids compared to PC after 1 h exposure. Deletion of the *bft-*2 gene from ETBF had no effect on OMV inhibition of CFTR Cl⁻ secretion compared to OMVs secreted by ETBF expressing BFT-2. NTBF+*bft* OMVs were significantly more inhibitory to CFTR Cl⁻ secretion than OMVs secreted by NTBF. OMVs were also non-toxic to colon organoids.

Twenty-four hours after exposure to OMVs, CFTR Cl⁻ secretion was reduced in ETBF, ETBF Δ*bft,* and NTBF+*bft* compared to PC. OMVs secreted by NTBF+*bft* significantly reduced CFTR Cl⁻ secretion compared to NTBF. Thus, *bft*-2 addition to NTBF reduced CFTR Cl⁻ secretion, but deletion of *bft-2* in ETBF had no effect on CFTR Cl⁻ secretion compared to ETBF. Thus, the ability of BFT-2 to reduce Cl⁻ secretion by colon organoids was strain-dependent. Since BFT-2 is a metalloprotease, we speculate that the effect of NTBF+*bft* OMVs on colon organoids may be related to the ability of BFT-2 to catalyze peptide bond hydrolysis. Since OMVs secreted by strains of *B. fragilis* that lacked *bft*-2 (BFT Δ*bft* and NTBF) also reduced CFTR Cl⁻ secretion at 1 h and 24 h post-OMV exposure, there must be other factors in *Bacteroides* OMVs, including, but not limited to, proteins or miRNAs, that inhibit forskolin-stimulated CFTR Cl⁻ secretion. For example, in previous studies on *Pseudomonas*, we demonstrated that OMVs contain Cif, a virulence protein, which reduced CFTR Cl⁻ secretion by inhibiting endocytic recycling of CFTR in human bronchial epithelial cells (44). AprA, another virulence factor secreted in *Pseudomonas* OMVs, also inhibited CFTR Cl⁻ secretion (45). 5′ tRNA-fMet halves in *Pseudomonas* OMVs also reduced IL-8 secretion by human bronchial epithelial cells (22). Additional studies are required to elucidate the cellular mechanism whereby OMVs secreted by *Bacteroides* reduce CFTR Cl⁻ secretion by colon organoids.

By contrast to results from human colon organoids, all OMVs secreted by *B. fragilis* inhibited CFTR Cl⁻ secretion by T84 cells to the same extent at 1 h post-OMV exposure. The reason for this difference between the effect of OMVs on T84 cells and human colon organoids is unknown, but it may be related to several factors, including, but not limited to, possible differences in the time-dependent effects of BFT-2 as well as differential expression of genes and signaling pathways between T84 tumor cells and primary human colon organoids. Additional studies are required to identify these factors.

The observation that OMVs inhibited CFTR Cl⁻ secretion by primary human organoids is interesting to note since infection with ETBF in animals and human subjects causes secretory diarrhea, which one might expect to be caused by increased CFTR Cl⁻ secretion, a primary driver of fluid secretion by the colon (13, 14). However, there is differential expression and regulation of ion transporter proteins along the length of the colon, as well as differential responses to drugs, hormones, and immune factors, along the length of the colon (13, 14). Accordingly, our results are applicable to the proximal human colon, and other segments of the colon may react differently to OMVs and BFT-2. It is the composite effect of *Bacteroides*, their OMVs, and BFT-2 on the gastrointestinal tract that determines the *in vivo* response to ETBF.

Our studies also did not observe a significant effect of OMVs on cytokine secretion by human colon organoids. Most notably, IL-8 secretion was not significantly increased by OMVs (Fig. 12), in contrast to studies using recombinant BFT-2 in cell lines reporting that recombinant BFT-2 increased IL-8 secretion (9, 15, 17–20). The reason for this difference between the effect of OMVs on colon cell lines and human colon organoids is unknown, but it may be related to several factors, including, but not limited to, the amount of recombinant versus native BFT-2 in OMVs and gene signaling pathways in colon tumor cell lines and human primary colon organoids. Studies have also shown that the effect of recombinant BFT-2 and OMVs on colon cell lines is dose-dependent, and it is likely that the concentration of BFT-2 in OMVs is less than the doses of recombinant BFT-2 utilized in studies on cell lines (11, 18). We conclude that biologically relevant levels of OMVs (21, 40) secreted by *B. fragilis* inhibit CFTR Cl⁻ secretion, but have no effect on amiloride-sensitive sodium reabsorption, claudin-2, E-cadherin, TER, or on cytokine secretion by primary human colonoids.

Fragipain was not detected in any of the OMV samples (Table S4). Fragipain is known to cleave BFT-2 into its active form (46); however, studies suggest that this protein is not required for cleavage of BFT-2 in antibiotic-treated mice (36). More specifically, fragipain is unnecessary when recombinant BFT-2 is mixed with the colon mucus of pathogen-free or germ-free mice, suggesting that the mucosal activation process may be more prevalent in gastrointestinal conditions containing increased mucus, such as CF (36). Notably, primary human colon organoids also secrete mucin (see Fig. 5). PBS is also able to facilitate the cleavage of BFT-2 into its active form and further suggests that fragipain is not required for BFT-2 activation (36).

In accordance with previous research, our proteomics data revealed that several TonB-dependent receptors are more abundant in OMVs secreted by ETBF compared to NTBF strains (Table S4) (47). TonB-dependent receptors are one part of outer membrane structures that are critical for the influx of nutrients via SusC and SusD outer membrane proteins, which were elevated in ETBF OMVs compared to NTBF OMVs (37). An increase in the abundance of these proteins suggests an enhanced need for ETBF to increase nutrient acquisition, thereby improving its ability to compete for similar niches that NTBF does not struggle to fill. Regardless, these results pose interesting questions for mechanistic follow-up experiments in the future.

Importantly, although several studies have shown that recombinant BFT-2 degrades E-cadherin and claudin-2 in cell lines (9, 15, 17, 19, 20), none of the OMVs tested using biologically relevant concentrations herein had any effect on the abundance of the tight junction protein claudin-2, the cell adhesion protein E-cadherin, or on the TER. Claudin-2 is an important component of the tight junction, and reductions in claudin-2 abundance decrease TER (48, 49). Several possible explanations may account for the difference in the TER, E-cadherin, claudin-2, and cytokine response of colon tumor cell lines versus primary human colon organoids. First, as noted above, the effects of BFT-2 are dose-dependent (18). OMVs secreted by ETBF and NTBF+*bft* contain biologically relevant levels of BFT-2. It is possible that the concentration of recombinant BFT-2 in previous studies was much higher than the amount of native BFT-2 in OMVs, although a comparison of concentrations is not possible given available data. Second, the duration of exposure to OMVs may be a factor. The literature reports that the effects of recombinant BFT-2 on E-cadherin and claudin-2 in cell lines required anywhere from 30 min to several hours of exposure (3, 9, 10). Our study was limited to a 1 h exposure, which has been long enough to produce significant effects in many previous studies with recombinant BFT-2 and colon cell lines (3, 9, 10).

There are a few other reports describing the development of monolayers of epithelial cells derived from healthy colon organoids (31, 32). Sen et al. (32) reported that the gene expression profile of human colon organoids was significantly different from the Caco-2 cell line, an immortalized cell line of human colorectal adenocarcinoma cells, which had a gene expression profile similar to the small intestine. They reported that the effect of *Bifidobacterium longum* on gene expression was not conserved between Caco-2 and colon organoids. Taglieri et al. (31) reviewed the literature on human colon organoids and noted that air-liquid interface culture increased differentiation of colon organoids. However, none of the other studies on colon organoids exposed them to OMVs or reported CFTR Cl⁻ secretion.

OMVs secreted by most strains of *B. fragilis* in the present study, except NTBF, inhibited forskolin-stimulated CFTR Cl⁻ secretion by the proximal colon and T84 cells after a 1 h exposure, which would reduce salt and fluid secretion by the colon. However, studies in animals report that recombinant BFT-2 induces secretory diarrhea (3, 6–8). One possible reason for the different effects of recombinant BFT-2 and OMVs containing BFT-2 is that the effect of BFT-2 is dose-dependent, and that recombinant BFT-2 levels in published studies may be higher than the abundance of BFT-2 in OMVs. As demonstrated in Fig. 3 and the Supplemental tables, OMVs contain many proteins in addition to BFT-2, which may influence CFTR Cl⁻ secretion by primary human colon organoids. Finally, it is important to note that there are other pathways that mediate transepithelial salt and water transport by the proximal colon, including electroneutral NaCl and fluid reabsorption mediated by the parallel operation of Na^+^/H^+^ and Cl⁻/HCO_3_⁻ (SLC26A3) exchangers. Since Ussing chamber studies only measure electrogenic transport, it is possible that recombinant BFT-2 (and endogenous BFT-2 in OMVs) may inhibit SLC26A3 and/or the Na^+^/H^+^ exchanger *in vivo* and thereby reduce electroneutral NaCl and fluid reabsorption by the colon. Such an effect may contribute to the ETBF-induced secretory diarrhea, despite the OMV reduction in electrogenic CFTR Cl⁻ secretion reported herein. Thus, extensive additional studies, beyond the goals of the present report, are required to determine if BFT-2 and OMVs inhibit Na^+^/H^+^ and Cl⁻/HCO_3_⁻ electrically neutral NaCl and fluid reabsorption in primary human colon organoids. Such studies would require the use of radioactive isotopes and chemical inhibitors of electrically neutral transporters and/or antisense experiments to selectively reduce electroneutral transporter expression.

We also did not observe an effect of any OMVs on the TER or epithelial tight junction or cell adhesion structure. In many, but not all, previous studies in colon cell lines, recombinant BFT-2 quickly (< 1 h) reduced the abundance of E-cadherin and the TER (9, 15, 17, 19, 20). Although the reason for this difference is unknown, one possible explanation for the different effects of recombinant BFT-2 and OMVs is that, as noted above, the effect of recombinant BFT-2 is dose- and time-dependent, and that recombinant BFT-2 levels in published studies may be higher than the abundance of endogenous BFT-2 in OMVs.

In contrast to studies using cell lines and recombinant BFT-2 (10, 50–52), we did not observe a significant increase in IL-8 secretion in primary human colon organoids exposed to OMVs. The lack of effect of BFT-2 and OMVs on IL-8 secretion in our study is consistent with the observation that OMVs did not reduce E-cadherin, an effect reported to be required for BFT-2 to stimulate IL-8 secretion (10, 50–52). OMVs did not affect the secretion of any of the 48 cytokines as determined by the MILLIPLEX MAP Human Cytokine/Chemokine 48-Plex cytokine assay. The reason for this difference is also unknown and requires additional studies.

There are a few limitations of our study. First, since there are many differences in the content of OMVs secreted by ETBF and NTBF, such as DNA, RNA, miRNA, proteins (this study), and other virulence factors (24, 53) in addition to BFT-2, it is possible that some of these factors may act alone or in concert with BFT-2 to inhibit CFTR Cl⁻ secretion. Second, we did not complement the *bft-2* gene in ETBF Δ*bft*, due in part to the technical challenge of complementation in our clinical strain. Third, we have shown that BFT*-2* expression in NTBF OMVs inhibits CFTR Cl⁻ secretion in colon organoids, but that deletion of *bft-2* in ETBF did not alter the effect of ETBF OMVs on CFTR Cl⁻ secretion. The reason for the different effects on BFT-2 in ETBF and NTBF OMVs was not investigated, requiring additional study.

Our study has numerous strengths. First, our experiments were conducted on well-differentiated human colon organoids from three healthy donors, which increase scientific rigor and reproducibility. Although for most experimental endpoints, colon organoids from each donor had a similar response to OMVs, in one donor of the three (24 h time point, donor 101A) there was no difference between CFTR Cl⁻ secretion to NTBF OMVs and NTBF + *bft* OMVs (Fig. 6). If our study was limited to this donor, our results and interpretation of the data would have been significantly different. This highlights one of the drawbacks of using cell lines obtained from a single donor. Second, since *B. fragilis* resides in mucus overlying colon organoids, OMVs containing BFT-2 are biologically relevant. Notably, the concentration of OMVs used in the present study was similar to levels observed in conditioned cell culture media and biological fluids (21, 40), and the same number of OMVs was used in all experimental groups (21, 40).

In conclusion, we have examined the effects of OMVs on well-differentiated primary cultures of human colon organoids secreted by *B. fragilis* containing *bft-2* (ETBF and NTBF + *bft*), a strain of *B. fragilis* in which *bft-2* was knocked out (ETBF Δ*bft*), and an NTBF strain that does not express *bft-2*. NTBF BFT-2–containing OMVs secreted by *B. fragilis* reduced forskolin-stimulated CFTR Cl⁻ secretion by colon organoids more than the same strain lacking *bft-2* but had no effect on tight junction structure (E-cadherin and claudin-2), TER, amiloride-sensitive sodium absorption, or IL-8 secretion. Similar experiments on the T84 cell line also revealed that OMVs secreted by *B. fragilis* reduced CFTR Cl⁻ secretion and had no effect on TER. We conclude that *B. fragilis* OMVs inhibit forskolin-stimulated CFTR Cl⁻ secretion by primary human colon organoids, without affecting TER, amiloride-sensitive Na^+^ currents, and cytokine secretion. Additional studies, beyond the scope of the present study, are required to elucidate the cellular mechanisms whereby OMVs rapidly inhibit CFTR Cl⁻ secretion, and why BFT-2 in NTBF OMVs reduces CFTR Cl⁻ secretion, compared to NTBF OMVs lacking *bft-2*, and why deletion of bft-2 in ETBF had no effect on Cl⁻ secretion. Although the results in this study contradict many reports using cell lines and recombinant BFT-2, we anticipate that our results will stimulate a discussion of the effects of BFT-2 on the colon.

## MATERIALS AND METHODS

### *Bacteroides* culture and genetics

*B. fragilis* NCTC 9343 was chosen as the non-toxigenic *B. fragilis* (NTBF) strain, since it is widely used (54–56). *B. fragilis* 86-5443-2-2 was chosen as the enterotoxigenic *B. fragilis* (ETBF) strain for similar reasons and because it expresses BFT-2, the most virulent of the three BFT isotypes (54–56). *B. fragilis* 86-5443-2-2 with a *bft* deletion (ETBF Δ*bft*), provided by Dr. Cynthia Sears, was confirmed by PCR (57). To construct the NTBF+*bft* strain, we first PCR-amplified *bft-2* from *B. fragilis* 86-5443-2-2 and cloned it via Gibson Assembly into the SalI and NcoI sites of the *Bacteroides* expression plasmid pNBU2-P1T-DP-A21 (58). This plasmid integrates in single copy into the chromosomal att sites of *Bacteroides fragilis*. The sequence of the resulting plasmid was confirmed by Sanger sequencing before transformation into *E. coli* S17-1 cells and conjugation via overnight aerobic solid agar co-culture mating into *B. fragilis* 9343 (NTBF). Following incubation, matings were resuspended and plated on BHIS agar containing gentamicin and erythromycin. The att chromosomal integration site was confirmed by PCR (59). *Bacteroides* strains were grown on supplemented brain heart infusion medium (BHIS) agar plates or in liquid broth cultures supplemented with hemin and 1 μg/mL of vitamin K3 (Acros Organics/Fisher Scientific, Geel, Belgium) under anaerobic conditions (10% CO_2_, 10% H_2_, 80% N_2_) in a Whitley A55 anaerobic chamber (Don Whitley Scientific, Victoria Works, UK) at 37°C. All BHIS agar and liquid media contained 60 μg/mL of gentamicin sulfate. For OMV purification, *B. fragilis* strains were streaked on BHIS agar plates and were grown in anaerobic conditions for 48 h. Single colonies were used to inoculate 5 mL of BHIS liquid medium cultures, which were grown anaerobically for 24 h. The cultures were then expanded into 20 0mL BHIS cultures using a 1:100 dilution. The final cultures were grown for 24 h prior to being utilized to isolate OMVs.

### Outer membrane vesicle (OMV) isolation

OMVs were isolated as described by us and others previously (23, 60). Briefly, all overnight culture supernatants were centrifuged for 1 h at 2,800 × *g* and 4°C to pellet bacteria. The supernatant was filtered twice through 0.45 μm PVDF membrane filters (Millipore, Billerica, MA, USA) to remove any contaminating bacteria and concentrated with 30K Amicon filters (Millipore, Billerica, MA, USA) at 2,800 × *g* and 4°C to obtain ~200 µL of concentrate. The concentrate was resuspended in OMV buffer (20 mM HEPES, 500 mM NaCl, pH 7.4) and subjected to ultracentrifugation using a TH-660 Swinging Bucket rotor (Thermo Fisher) for 2 h at 39,000 × *g* and 4°C to pellet OMVs. OMV pellets were then re-suspended in 60% OptiPrep Density Gradient Medium (Sigma-Aldrich, Cat# D1556) and layered with 35%, 30%, and 20% OptiPrep diluted in OMV buffer. OMVs in OptiPrep were centrifuged for 18 h at 100,000 × *g* and 4°C. Fractions of 500 μL were taken from the top of the gradient, with OMVs residing in fractions 5–8, as determined by proteomic analysis of OMVs.

### Electron microscopy of OMVs

OMVs were visualized with negative staining transmission electron microscopy as described previously (22). The microscopist was blinded to the experimental treatment to minimize observer bias. Briefly, an aliquot of each sample was applied onto a freshly glow-discharged 200-mesh nickel grid and allowed to sit for 2 min. After incubation, excess fluid was gently removed with filter paper, and the grids were sequentially rinsed with Millipore-filtered distilled water. While the grids remained slightly damp, they were placed onto a drop of Nano-W tungsten stain (Ted Pella, Inc.). Excess stain was absorbed with filter paper, and the staining step was repeated on a second drop of Nano-W, which was allowed to sit for 1 min before being wicked away and left to dry. Imaging of the stained grids was conducted at 80 kV on a JEOL 1400 transmission electron microscope (JEOL USA Inc.), and images were captured using an AMT XR11 digital camera (AMT).

### Proteomics characterization of OMVs

To confirm that BFT-2 was secreted by ETBF and NTBF+*bft* in OMVs, but not by ETBF Δ*bft* and NTBF OMVs, OMVs were isolated as described above, pelleted, and lysed in buffer containing 0.5% SDS, 50 mM ammonium bicarbonate, 50 mM NaCl, and Halt Protease Inhibitor. Mass spectrometry analysis was performed by the Mass Spectrometry Technology Access Center (MTAC) at Washington University School of Medicine using previously published protocols (61). Samples were purified by trichloroacetic acid precipitation. After pelleting and washing with ice-cold acetone, the resulting protein pellet was resuspended in 8 M urea and 0.4 M ammonium bicarbonate, reduced with 4 mM dithiothreitol, and alkylated with 18 mM iodoacetamide. The solution was then diluted to <2M urea, and 1 µg of trypsin was added for overnight digestion at 37°C. The resulting peptides were desalted using C18 solid-phase extraction spin columns, and eluates were dried under vacuum using a SpeedVac concentrator. Peptides were analyzed by LC–MS/MS using a nanoElute2 coupled to a timsTOF Pro2 mass spectrometer (Bruker Daltonics). Samples were separated on a C18 column (150µm × 25 cm, 1.5 µm; Bruker). The mobile phases consisted of 0.1% formic acid (FA) in water (mobile phase A) and 0.1%FA in acetonitrile (mobile phase B). Peptides were separated using a 54 min linear gradient from 3% to 35% mobile phase B, with a total run time of 60 min at a constant flow rate of 600 nL/min. The timsTOF Pro2 was operated in dia-PASEF mode. Spectra were acquired across an m/z range of 300–1,200, using 28 mass windows per cycle. The inverse reduced ion mobility (1/K₀) range was set to 0.65–1.35 V·s/cm². The trapped ion mobility spectrometry (TIMS) analyzer operated at a 100% duty cycle with a ramp time of 75 ms, yielding an overall cycle time of approximately 1.20 s. Collision energy was linearly ramped from 59 eV at 1/K₀ = 1.6 to 20 eV at 1/K₀ = 0.6. dia-PASEF data files were analyzed using directDIA workflow embedded in Spectronaut 18 (Biognosys AG) against the *Bacteroides fragilis* database. Search parameters included trypsin digestion with cleavage after K or R, allowance for up to two missed cleavages, carbamidomethylation of cysteine (static modification), and variable modifications, including oxidized methionine and protein N-terminal acetylation. Peptide spectrum matches (PSMs), peptides, and protein groups were identified using a false discovery rate (FDR) threshold of 0.01. For quantification, all fragment ions passing the FDR threshold were used, and protein abundances were calculated as the area under the curve of extracted ion chromatograms within the defined peak boundaries for each targeted ion. The mass spectrometry proteomics data have been deposited to the ProteomeXchange Consortium via the PRIDE partner repository (62) with the data set identifier PXD073462.

### Colon organoid cell culture

Biopsies from the proximal colon, obtained during colonoscopies of three healthy de-identified, donors, were obtained after informed consent at Dartmouth Health. Biopsies were placed in PBS (−/−) with EDTA on a shaking rocker at 4°C for 1 h to release the crypts from the biopsies. Then, the biopsies were vigorously pipetted up and down several times to release the crypts. The supernatant containing crypts was collected, and additional PBS was added to the biopsies, followed by vigorous pipetting. Crypts in the supernatant were counted, pelleted, and resuspended in 55% Matrigel (Corning Life Sciences) and 45% isolation medium. Cells were added to 24-well plates, and then Matrigel-solidified cell isolation medium (IntestiCult complete medium with primocin [StemCell Technologies] supplemented with 50 µg/mL gentamycin, 50 µg/mL vancomycin, and 10 µM of RhoKi) was added to each well. Cells were maintained in culture until spherical organoids were observed. To develop polarized monolayers of organoids, 1.5 × 10^6^ cells (as spherical organoids) were collected from the Matrigel and seeded on Transwell permeable filters (#3450: Corning Life Sciences for 24 mm Transwell) or 12 mm Snapwell permeable filters (#3801; Corning Life Sciences). Filters were precoated with a 1:20 dilution of Matrigel in cold DPBS for 2 h and then removed just before seeding cells. IntestiCult medium (StemCell Technologies) was added to both apical and basolateral sides of the filters to support cell growth. Once a confluent monolayer was obtained, IntestiCult differentiation medium (StemCell Technologies) was used to differentiate the cells prior to experimentation. Cells were differentiated for one week before experiments.

### Exposure of primary human colon organoids to OMVs

Polarized monolayers of colon organoids on 12 mm Snapwell filters were washed with PBS to remove excess mucus, and 1.5 mL of differentiation medium was added to the basolateral side. 2 × 10^10^/mL OMVs from all strains of *B. fragilis*, a concentration of OMVs similar to that measured in conditioned media and in biological fluids (21, 40), or the same volume of process control (PC) media were applied to the apical side of cells for 1 h and then removed. At the end of the 1 h exposure and again 24 h later, the apical and basolateral media were collected and stored at −80°C until processing for cytokine analysis using the MILLIPLEX Human Cytokine/Chemokine/Growth Factor Panel A (48 plex, MilliporeSigma) in the DartLab Immunoassays Core according to the manufacturer’s recommendations. All samples were analyzed at the same time to minimize batch effects. Briefly, as stated in the MilliporeSigma website, “Calibration curves from reconstituted cytokine standards were prepared with fourfold dilution steps in the same matrix as the samples. High and low controls from the Millipore kits were included. Standards and controls were measured in triplicate; samples were measured once; and blank values were subtracted from all readings. All assays were carried out directly in a 96-well plate (Millipore) at room temperature and protected from light.

### Cytotoxicity

To determine if OMVs were cytotoxic to colon organoids, LDH release into the basolateral media was measured 1 h and 24 h after cells were exposed to OMVs using the Promega CytoTox 96 Non-Radioactive LDH Assay (Cat# G1780) according to the manufacturer’s instructions. Samples were stored at −80°C until LDH measurements. All samples were analyzed at the same time to minimize batch effects.

### T84 cell culture

T84 cells, a human colon adenocarcinoma cell line (American Type Culture Collection, Manassas, VA), were maintained in DMEM/F-12 media (ATCC) containing L-glutamine supplemented with 5% fetal bovine serum (FBS: Life Technology). For short-circuit current and TER measurements, cells were cultured on Transwell filters (Corning, Ithaca, NY). Experiments were performed 21 days post-seeding, as previously described (41–43).

We performed Ussing chamber measurements to assess sodium transport and CFTR Cl^−^ currents. As described in detail elsewhere (63–66), cells on Snapwell filters were mounted in Ussing chambers. Primary human colon organoids and T84 cells were exposed to either process control (PC: BHIS growth media processed through the OMV isolation procedure, as recommended by the International Society of Extracellular Vesicles) (67) or OMVs secreted by ETBF, ETBF Δ*bft*, NTBF, or NTBF+*bft* (all at 2 × 10^10^/mL), which were added to the apical side of monolayers for 1 h. Then, CFTR Cl⁻ currents, amiloride-sensitive sodium currents (in colon organoids only, since T84 cells do display amiloride-sensitive sodium currents), and TER were measured. Some monolayers were exposed to OMVs for 1 h, followed by washing OMVs from the apical surface, and 24 h later CFTR Cl⁻ currents and TER were measured. To measure TER, a 1 mV pulse was applied to each monolayer, the change in short-circuit current was measured, and TER was calculated using Ohm’s law. Electrogenic sodium currents were reported as amiloride-sensitive currents, since amiloride inhibits the epithelial sodium channel ENaC (68). To measure CFTR Cl⁻ secretion, amiloride (50 μM) was first added to the apical solution to inhibit and measure sodium reabsorption. Thereafter, Cl⁻ secretion was stimulated with forskolin (10 μM; Sigma-Aldrich), which increases protein kinase A, leading to phosphorylation and activation of CFTR Cl⁻ secretion (39). Finally, thiazolidinone (CFTR_inh_-172, 20 μM; Millipore, Billerica, MA), an inhibitor of CFTR Cl⁻ channels, was added to the apical solution (64). Data are expressed as the forskolin-stimulated, CFTR_inh_-172-inhibited short-circuit current (Isc), which is presented as μA/cm^2^. Data were collected and analyzed using the Data Acquisition Software, Acquire and Analyze program (Physiologic Instruments, San Diego, CA).

### Cytokine measurements

Cytokine secretion (apical and basolateral solutions) from primary human colon organoids was measured using the MILLIPLEX MAP Human Cytokine/Chemokine 48-Plex cytokine assay (MilliporeSigma) following the manufacturer’s instructions.

### Immunocytochemistry

Immunocytochemistry was performed to characterize cell types in the colon organoids and to evaluate the effects of OMVs on E-cadherin and claudin-2. Fixation of colon organoids was performed using 4% paraformaldehyde for 15 min at room temperature, followed by permeabilization with 0.25% Triton X-100 for 15 min, blocking with 10% normal goat serum in PBS, and primary antibody incubation at 4°C overnight. After washing, secondary antibodies were added for 1 h at room temperature in the dark. SLC26A3 was labeled with a primary anti-mouse antibody (1:250, Santa Cruz #sc-376187) and a secondary goat anti-mouse-488 antibody (1:500, Santa Cruz #sc-376187). CFTR was labeled with a primary anti-mouse antibody (1:100, Millipore # 05-583) and a secondary anti-mouse-488 antibody (1:500). MUC2 was labeled with a primary anti-mouse antibody (1:300) and a secondary anti-mouse-488 antibody (1:500, Abcam ab11197). Chromogranin A was labeled with a primary goat anti-rabbit antibody (1:100) and a secondary goat anti-rabbit-568 antibody (1:1000, Abcam ab15760). Tight junction labeling was performed by fixing cells in 100% cold methanol at −20°C for 5 min, blocking with 10% goat serum in PBS for 1 h, and incubating with primary antibodies at 4°C overnight. The secondary antibody was added for 1 h at room temperature the following day. Claudin-2 was labeled using a primary goat anti-rabbit antibody (1:100, Thermo Fisher #51-6100) and a secondary goat anti-rabbit-568 antibody (1:500). E-cadherin was labeled using a primary goat anti-mouse antibody (1:250, BD Transduction lab #610,182) and a secondary goat anti-mouse-488 antibody (1:500). All antibodies were diluted in PBS containing 5% normal goat serum. DAPI staining to label nuclei was performed using Fluoroshield anti-fade mounting medium. To image the colon organoids, a Yokogawa W1 spinning disk unit coupled to a Nikon Ti-E inverted microscope was used. To examine the effect of OMVs and PC on claudin-2 and E-cadherin, all settings on the confocal microscope were identical for acquiring images for analysis. Analysis of claudin-2 and E-cadherin abundance was performed by an investigator blinded to the treatments. The membrane areas with positive fluorescence signal were segmented using Trainable WEKA machine learning plugin in ImageJ (69). The segmented objects were thinned to one pixel using ImageJ’s “Skeletonize” morphological operation. The mean and median fluorescence intensities were measured from areas defined by the skeleton and widened to five pixels using ImageJ’s “Dilate” morphological operation.

### Statistics

Data were analyzed using the R software environment for statistical computing and graphics version 4.1.0 (70), Ingenuity Pathway Analysis, and Prism (GraphPad). Statistical significance was calculated using mixed-effects linear models or one-sample *t*-tests, as indicated in the figure legends. Mixed-effects linear models were used instead of standard one-way ANOVA when donors were measured across multiple treatment conditions, creating a correlated (blocked) data structure. Modeling donor as a random effect appropriately accounts for this within-donor correlation and separates donor-to-donor variability from treatment effects. For PC-adjusted ΔIsc data (Fig. 8), where the outcome is already expressed as a difference from the process control, one-sample *t*-tests against zero were used to assess whether each treatment differed from PC, with technical replicates averaged within donor prior to analysis.

Data were visualized, and figures were created using Prism or ggplot.

## Data Availability

The mass spectrometry proteomics data are available in the Table S1 and have been deposited in the ProteomeXchange Consortium via the PRIDE partner repository (62) with the dataset identifier PXD073462. The URL is https://ftp.pride.ebi.ac.uk/pride/data/archive/2026/05/PXD073462. All other reasonable requests for additional data are available from the corresponding author.

## SUPPLEMENTAL MATERIAL

Supplemental tables iai.00129-26-s0001.xlsx Tables S1 to S4.

ASM does not own the copyrights to Supplemental Material that may be linked to, or accessed through, an article. The authors have granted ASM a non-exclusive, world-wide license to publish the Supplemental Material files. Please contact the corresponding author directly for reuse.
